# Diagnosis of mycobacterial drug resistance in HIV reactive patients by phenotypic and genotypic assay - a comparative study

**DOI:** 10.1186/1471-2334-14-S3-P58

**Published:** 2014-05-27

**Authors:** S Umamaheshwari, Sumana M Neelambike, K Anuradha, B Sumangala

**Affiliations:** 1 Department of Microbiology, JSS Medical College, Mysore, Karnataka, India; 2 Department of Microbiology, Mysore Medical College and Research Centre, Mysore, Karnataka India; 3 Department of Microbiology, Mandya Institute of Medical Sciences, Mandya, Karnataka, India

## Background

Emergence of drug resistance has complicated the treatment of Tuberculosis (TB). WHO reports India as one among 27 “high burden” multidrug resistant (MDR) TB countries. The study was carried out to detect drug resistance of mycobacterial isolates in HIV patients.

## Methods

The clinical specimens collected from HIV seropositive AFB smear negative patients (Group A) with features of TB, AFB smear positive, HIV reactive (Group B) and AFB smear positive, HIV negative (Group C) were processed as per standard protocol. They were inoculated onto Lowenstein Jensen media and the isolates obtained were subjected to drug susceptibility test (DST) by proportion method and Genotype MTBDR plus assay.

## Results

In Group A out of 162 patients, 443 clinical samples were collected and 76 mycobacterial strains were obtained from 67 (41%) patients. In this 69 (91%) were *Mycobacterium tuberculosis* complex and 7 (9%) were *Mycobacterium avium* complex. As per phenotypic method, of 76 isolates, 50 (65.8%) were sensitive to all drugs and 26 (34.2%) resistant to one or more drugs. The MDR and resistance to at least single drug of Group A, B and C were [5 (6.6%), 2 (6.7%), 4 (8%)] and [26 (34.2%, 3(10%), 8 (16%)] respectively. MTBDR assay showed similar results except one discrepancy i.e., one isolate was resistant to rifampicin that was sensitive in proportion method.

## Conclusion

The increase of MDRTB in high TB burden settings stresses the need for DST. This helps in providing proper treatment regimens, improve prognosis and prevent the spread of drug resistant TB.

